# Diagnosis and surgical treatment of carotid body tumor: A report of 18 cases

**DOI:** 10.4103/0975-3583.70905

**Published:** 2010

**Authors:** Dan Ma, Min Liu, Hua Yang, Xiaogan Ma, Chaojun Zhang

**Affiliations:** *Department of General Surgery, Xinqiao Hospital, Third Military Medical University, Chongqing 400037, P. R. China*

**Keywords:** Carotid body tumor, diagnosis, treatment

## Abstract

**Objective:**

To summarize the experience in the diagnosis and treatment of carotid body tumor (CBT).

**Materials and Methods:**

CBT in 18 cases was confirmed by digital subtraction angiography (DSA). Resection of the tumor under the carotid adventitial plane was performed in 10 cases, the tumor with the external carotid artery in five cases, and the tumor with the internal and external arteries at the same time in three cases.

**Results:**

Neither death nor any major complications occurred in all the 18 cases. Our follow-up of the 18 patients revealed neither recurrence nor metastasis.

**Conclusion:**

DSA is the gold standard for the diagnosis of CBT. After confirmation, thorough preoperative examination, sufficient preoperative preparation, and correct surgical approaches can result in satisfactory surgical effects.

## INTRODUCTION

Carotid body tumor (CBT) is one of the most commonly seen jugular paraganglioma involving the carotid body chemoreceptors, but rarely seen clinically, so the corresponding diagnosis and management remain difficult. Eighteen patients with CBT were admitted into our department from 1997 to 2008. We would like to investigate the experience in the diagnosis and management of CBT through a retrospective analysis of the clinical data.

## MATERIALS AND METHODS

### Patient characteristics

**Figure 1:**
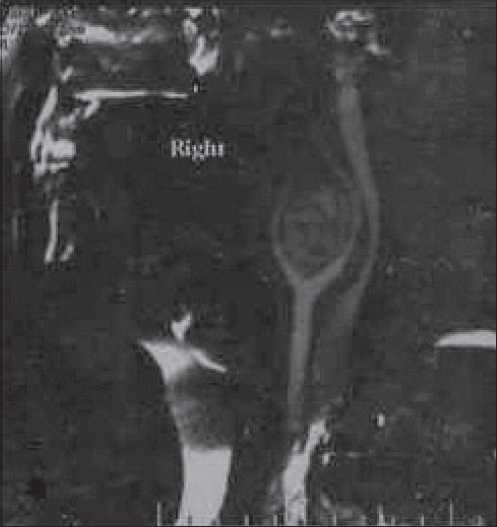
CT scan of neck reveals a tumor mass at the bifurcation of the right carotid artery

Eighteen cases (male: 10, female: 8, age range: 28–66 years; average age: 52) were employed in our report. Lateral lesion was found in 17 cases, bilateral in 1 case, left in 13 cases, and right in 5 cases. Hospital visit due to painless masses under the mandibular angle was found in 18 cases complicated with hoarseness in one case and bradycardia and faint in one case. Color Doppler sonography and DSA were performed in all patients for the confirmation. Additional spiral CT angiography (SCTA) was performed in patients with the disease history for near 2 years and imaging 3D reconstruction was also performed. A tumor mass locate at the bifurcation of the carotid artery [Figure 1]. Besides, additional CT scan of the mediastinum and retroperitoneal sonography were also performed in patients with family history and multiple foci of disease. For the purpose of improvement of the cerebral anoxia tolerance and compensation, preoperative training for the carotid arterial pressure was performed in all patients, lasting from 10 min to 30 min every time, 3–5 times daily. After no obvious spinning sensation, the patients underwent transcranial Doppler assessment of the cerebral bypass circuit with the bilateral difference less than 30% as the standard. In addition, preoperative ultraselection embolism of the feeding artery was performed in five cases.

### Surgical technique

Under general anesthesia, laterally inclined incision along the anterior border of the sternomastoid muscle was performed intraoperatively. After clear visualization of the anatomic structures such as the common carotid artery, the internal carotid vein, the cranial nerve, and the accessory nerve, then the common carotid artery and the internal and external carotid veins were sufficiently liberated for a good separation, and then the common carotid artery and the proximal end of the tumor artery were blocked, using blood vessel blocking bands for the convenient control of the blood flow, and finally they were carefully separated along the tumor body so that the blood vessels feeding the tumor could be radically removed. Separation was performed in 10 cases with relatively separated tumor body from the artery under the carotid adventitial plane. It was impossible to perform separation due to tight adherence of the tumor body to the artery in eight cases, out of which resection of the tumor body and the external carotid artery were performed in five cases, resection of the tumor body, and the internal and external arteries in three cases. Reconstruction of the common carotid and the internal carotid artery were performed in patients undergoing resection of the internal carotid artery. The minimum and maximum sizes of the specimens in 18 cases were 2.0 cm × 1.8 cm × 1.5 cm and 7.0 cm × 6.5 cm × 5.5 cm, respectively. All specimens were confirmed pathologically as carotid artery tumor (jugular paraganglioma).

## RESULTS

Neither death from operation, blood flow was easy and smooth after carotid artery repair. No cerebral ischemia was observed after operation. No nerve injuries occurred in all the 18 cases. However, preoperative hoarse voice partially recovered after the operation. A follow-up from 11 months to 11 years found neither recurrence nor metastasis in all cases.

## DISCUSSION

The mechanisms of CBT, as the most commonly seen type of jugular paraganglioma, remain unknown, but hypoxia and genetic factors are thought to be involved in the pathogenesis of this disease. Related studies have proved that disease-induced low partial pressure of the oxygen in the blood and chronic continuous hypoxia of people living in high altitudes (above 1500 m) and chronic intermittent hypoxia due to sleep apnea syndrome could stimulate the hyperplasia and hypertrophy of the carotid glands. In CBTs, genetic-type amounts to approximately 35%, in which higher disease incidence of multiple ganglioneuroma is found, and if individuals with this type of gene mutations experience chronic hypoxia, then tumor may develop at the early age, but under the condition of no family history, predisposing genes can spontaneously emerge, inducing CBTs.[1] CBT is often seen in patients at the age from 50 to 70 years old with higher incidence in female than that in male. Most patients receive medical treatment for accidental finding of the transverse masses in the cervical part, and hence some patients may complain of such symptoms as local tremor or pulse-like vibratory sense in the mass site, and headache, change in voice, vertigo, etc. Involved nerves can lead to the corresponding symptoms in the nerve-dominated area, but symptoms mediated by endocrine changes are rare.[2] Physical checkup can find typical transverse beating masses, characterized by high-transverse mobility but low longitudinal mobility. The 8th to the 12th cerebral nerves involved may lead to the corresponding signs, but the *mandibular ramus* of the 7th nerve is rarely involved. According to the currently applied Shamblin’s classification, CBTs, based on the relationship between the mass and the carotid artery wall, are classified into three types: Type I, referring to those without encasement of the vessel wall, tumor size <5 cm, no widened carotid bifurcation, and easy for surgical removal; Type II, referring to those attached to the blood wall, but without encasement; Type III, referring to those located inside the blood vessel with encasement of the blood wall, tumor size larger than 5 cm with widened carotid bifurcation. Color Doppler Sonography and digital subtraction angiography (DSA) play a very important role in confirmation of the clinical diagnosis of CBTs, and DSA is regarded as the gold standard for the final diagnosis of CBTs. With the rapid development of CT technology, SCTA can facilitate the 3D-reconstructed image that can help demonstrate more directly the relationship of the tumor with the surrounding tissues. DSA cannot only provide us with information such as intracranial and extracranial blood circulation and Shamblin’s classification but also opportunity for embolization of blood vessels, resulting in decrease in intraoperative blood loss by occlusion of the blood vessels feeding the tumor through ultraselection arterial embolism.[3,4] Some researchers recently reported that preoperative application of covered stents for blocking the blood vessels feeding the tumor could achieve satisfactory therapeutic effects.[5] For tumors classified as Shamblin II and III, balloon occlusion test of the intracranial arterial blood vessel besides angiography could also be conducted to make sure whether the patient could tolerate this kind of blood vessel ligation or removal. If patients have the history of neural tumor, family history, or multiple foci of disease, whether or not multiple lesions should be taken into consideration, and hence it is indispensable to perform preoperative CT scan of the mediastinum and retroperitoneal sonography. Although surgical resection is the optimal choice for the treatment of CBTs, such conditions are listed as contraindications: (1) tumors damaging preoperatively the lateral cranial nerve or ganglionated cord, or removed surgically; (2) as to lesions classified as Shamblin III, radiotherapy should be the optimal choice for elderly patients or patients with chronic diseases because of the potential serious operative injuries and stroke-induced by the operation.[6] The intraoperative separation of the tumor body should be started at the site where the common carotid artery is lightly attached to the internal and external carotid arteries and should be centered on the bifurcation to manage the tumor-surrounding tissues and finally the bifuration. To prevent from hard control of bleeding due to cracked tumor body, measures for the control of bleeding should be carefully prepared before separation: blood vessel ligation band as well as atraumatic hemostatic forceps. While separation is in progress, the vessels feeding the tumor have to be ligated completely. Preoperative ultraselection arterial embolism of the feeding artery could result in obvious decrease in bleeding. All the 10 patients undergoing resection of the tumor under the external coat of vessels could be classified into Shamblin I, with smaller tumor body (the maximal diameter in this group was less than 3 cm). The minimal diameter of the tumor in five patients (Shamblin II) undergoing resection of the tumor and the external carotid artery and in three patients (Shamblin III) undergoing resection of the tumor as well as the internal and external carotid arteries was larger than 3.5 cm. Malignant CBTs amounted to 5–7%, higher occurrence rate in young patients and those with family history. The final diagnosis of malignant CBTs could be made, depending on recurrence and metastasis, partial recurrence combined with pains and lesions in the involved nerves, and distant metastasis involving organs such as bones, liver, and lungs. The reported postoperative metastasis emerged in 20 years suggests long-term followup should be insisted. Surgical removal is still the optimal option for the treatment of metastatic CBTs. Radiotherapy can effectively prolong the survival period of patients who cannot undergo surgery, but the chemotherapeutic effects remain unclear.[7]
